# A processed dietary pattern is associated with gestational diabetes mellitus and altered APOM and SAA4 proteomic signatures in Miao women

**DOI:** 10.3389/fnut.2026.1933838

**Published:** 2026-10-28

**Authors:** Wansong Zhang, Chenyu Liu, Yuan Sun, Danqing Zhao, Liming Shen, Xiaorong Ni, Tian Qiao, Ying Zong, Yi Liang

**Affiliations:** 1 Department of Comprehensive, Quality Management Office, Affiliated Hospital of Guizhou Medical University, Guiyang, Guizhou, China; 2 Department of Medical, Guizhou Hospital Affiliated to the First Affiliated Hospital of Sun Yat-sen University, Guiyang, Guizhou, China; 3 Key Laboratory of Environmental Pollution Monitoring and Disease Control, Ministry of Education, School of Public Health, Guizhou Medical University, Guiyang, Guizhou, China; 4 Collaborative Innovation Center for Prevention and Control of Endemic and Ethnic Regional Diseases Co-constructed by the Province and Ministry, Guizhou Medical University, Guiyang, China; 5 College of Life Science and Oceanography, Shenzhen University, Shenzhen, Guangdong, China; 6 Department of Obstetrics, Affiliated Hospital of Guizhou Medical University, Guiyang, Guizhou, China; 7 Department of Clinical Nutrition, Shenzhen Hengsheng Hospital, Shenzhen, Guangdong, China; 8 Department of Clinical Nutrition, Affiliated Hospital of Guizhou Medical University, Guiyang, Guizhou, China

**Keywords:** dietary intake, differential proteins, food frequency questionnaire, gestational diabetes mellitus, matched case–control study, proteomics

## Abstract

**Background:**

This study integrated dietary pattern analysis with serum proteomics to identify diet-related molecular signatures associated with gestational diabetes mellitus (GDM) in Miao pregnant women.

**Methods:**

A 1:1 matched case–control study was conducted among 78 Miao pregnant women (39 GDM cases and 39 controls). Dietary patterns were derived using principal component analysis. Serum proteomics was performed using data-independent acquisition mass spectrometry. Associations were evaluated using conditional logistic regression and linear mixed-effects models. LASSO penalized logistic regression was used for exploratory feature selection, followed by nested cross-validation to assess internal discriminative performance and an exploratory multivariable analysis.

**Results:**

Higher adherence to a processed dietary pattern was associated with higher odds of GDM (OR = 2.33, 95% CI: 1.08–5.04). Proteomic analysis identified 34 candidate differentially expressed proteins enriched in lipid metabolism and inflammatory pathways. The processed dietary pattern was inversely associated with APOM (β = −0.308, *p* = 0.036) and showed a borderline inverse association with SAA4 (*β* = −0.285, *p* = 0.052). A leave-one-matched-pair-out nested LASSO feature-selection analysis using all 344 quality-controlled proteins yielded an internal AUC of 0.953 (95% CI: 0.894–0.992). SAA4 was selected in all 39 outer iterations, whereas selection frequencies varied across other proteins. APOM was retained for subsequent exploratory integrative analysis based primarily on its association with the processed dietary pattern, PPI-hub status, and biological relevance.

**Conclusion:**

A processed dietary pattern was associated with GDM and altered circulating APOM/SAA4 profiles in Miao pregnant women. Nested cross-validation provided an exploratory assessment of proteomic feature stability using all 344 quality-controlled proteins. The resulting internal discrimination should be interpreted cautiously because the upstream QC correction/normalization incorporated GDM group information and was completed before cross-validation. The APOM/SAA4–diet model was developed as a proof-of-concept analysis only and should not be interpreted as a clinically applicable prediction model. Larger independent and prospective cohorts are required for external validation.

## 1 Introduction

Gestational diabetes mellitus (GDM), defined as glucose intolerance first recognized during pregnancy, represents one of the most prevalent obstetric complications worldwide, affecting approximately 14.0% of pregnancies globally per IADPSG diagnostic criteria (1). GDM confers a range of adverse perinatal outcomes — including preterm birth, cesarean delivery, pre-eclampsia, macrosomia, and neonatal hypoglycemia (2, 3). It causes substantial long-term metabolic consequences for both mother and offspring: an approximately ten-fold elevated maternal risk of type 2 diabetes mellitus (T2DM), and heightened susceptibility to obesity and metabolic dysfunction in offspring (4–6). The escalating prevalence of GDM, driven by rising rates of obesity, advanced maternal age, and sedentary lifestyles, underscores the need for improved strategies for early risk identification and preventive intervention (7).

Current GDM diagnosis relies primarily on the oral glucose tolerance test (OGTT) administered at 24–28 weeks of gestation, despite evidence that clinically relevant metabolic disturbances may precede this window (8, 9). Given these limitations, identifying reliable biomarkers for early GDM prediction and timely intervention remains a clinical priority (10, 11). Within this prevention-oriented framework, dietary behavior has emerged as a particularly tractable modifiable target for GDM risk reduction (12–14).

Dietary patterns, which capture the synergistic effects of multiple foods consumed in combination, offer greater biological relevance than single-nutrient approaches in assessing GDM risk (15). Nutritional status is recognized as a key contributor to GDM, though the precise role of specific maternal dietary patterns during pregnancy remains incompletely understood (12, 16). Epidemiological evidence consistently links dietary pattern quality to GDM risk: Mediterranean, prudent, and vegetable-predominant patterns are each inversely associated with GDM (pooled RRs of 0.71, 0.78, and 0.86, respectively). Conversely, Western dietary patterns which are characterized by high intakes of red and processed meat have been linked to increased GDM risk (pooled RR = 1.27, 95% CI: 1.03–1.56; red meat: RR = 1.94, 95% CI: 1.41–2.67; processed meat: RR = 1.50, 95% CI: 1.16–1.94) (12, 17, 18). Plant-based (RR = 0.88, 95% CI: 0.81–0.96) and vegetable-fruit-dominant (OR = 0.33, 95% CI: 0.15–0.74) dietary patterns are associated with more favorable glycemic outcomes, plausibly through modulation of insulin sensitivity, gut microbiota composition, and systemic inflammation (19–21). Nevertheless, the molecular mediators underlying these diet–GDM associations have yet to be systematically elucidated (16).

Proteomics is a large-scale characterization of the protein complement expressed in a biological system that offers a powerful platform for interrogating the molecular underpinnings of GDM (22). Unlike the relatively stable genome, the proteome is highly dynamic and context-dependent, reflecting ongoing physiological and pathological processes (23–25). Mass spectrometry-based proteomics has identified 262 candidate GDM biomarkers, with the most replicable enriched in complement and coagulation cascades; exosomal and serum proteomic analyses further implicate immune and inflammatory dysregulation in early GDM (26–28). Collectively, these studies establish proteomics as a sensitive approach for GDM risk stratification and biomarker discovery.

A critical but underexplored frontier lies at the intersection of dietary patterns and the circulating proteome. Dietary patterns have been shown to induce broad alterations in circulating proteins with dietary-pattern-associated proteins enriched in pathways related to metabolism, immunity, and inflammation (16, 29). Integrating proteomic profiling with dietary pattern analysis in GDM could therefore identify protein mediators linking dietary exposure to metabolic risk and inform precision dietary strategies for prevention (30). Yet systematic studies examining the proteomic correlates of dietary patterns in pregnant women with or at risk for GDM remain scarce.

The present study employs a matched case–control design integrating dietary pattern assessment with serum proteomic profiling in pregnant women, aiming to characterize the proteomic signatures associated with distinct dietary patterns and their relationship with GDM risk. This approach provides a molecular basis for evidence-based nutritional recommendations in obstetric care.

## 2 Materials and methods

### 2.1 Study design and clinical sample collection

This study was designed as a case–control study. Participants were recruited from Miao pregnant women who received routine antenatal care and examinations between March 2023 and January 2025 at three tertiary hospitals in Kaili City, Qiandongnan Miao and Dong Autonomous Prefecture, Guizhou Province, China, including Qiandongnan Prefecture People’s Hospital, Kaili Maternal and Child Health Hospital, and the Second Affiliated Hospital of Guizhou Medical University. This study was conducted in accordance with the principles of the Declaration of Helsinki. The research protocol was reviewed and approved by the Ethics Committee of the Affiliated Hospital of Guizhou Medical University (Approval No. 2021–065-1). Written informed consent was obtained from all participants prior to their enrollment in the study.

Eligible participants were women aged ≥18 years, with singleton pregnancy, gestational age ≤28 weeks (based on the last menstrual period and confirmed by first-trimester ultrasonography), self-reported Miao ethnicity, and residence in Qiandongnan Prefecture for at least 1 year. Women were excluded if they had pregestational diabetes or fasting glucose ≥7.0 mmol/L before pregnancy, a previous diagnosis of gestational diabetes mellitus (GDM) in earlier pregnancies, severe hepatic or renal disease, malignant tumors, autoimmune disorders, or long-term use of medications that might affect glucose metabolism, such as glucocorticoids.

Between 24 and 28 gestational weeks, all participants underwent a standard 2-h 75 g oral glucose tolerance test (OGTT) for GDM screening. GDM was diagnosed according to the 2011 diagnostic criteria issued by the Ministry of Health of China, namely fasting serum glucose ≥ 5.1 mmol/L, 1-h serum glucose ≥10.0 mmol/L, or 2-h serum glucose ≥8.5 mmol/L, with fulfillment of any one criterion considered diagnostic. Ultimately, 39 women with newly diagnosed GDM were included as cases, and 39 women with normal glucose tolerance were selected as controls by 1:1 individual matching on age, gestational age, and body mass index (BMI), generating a unique matched-pair identifier.

Participants were assessed at two prespecified antenatal time windows: 10–12 gestational weeks and 24–28 gestational weeks (The day before the OGTT). At each scheduled visit, the dietary assessment using the FFQ and fasting venous blood collection were performed on the same day, thereby ensuring temporal concordance between dietary assessment and biomarker sampling. The FFQ assessed habitual dietary intake during the preceding trimester or the corresponding gestational period. Fasting venous blood samples were collected in the morning. Blood samples were allowed to stand at room temperature for 1 h and then centrifuged at 3000 rpm for 10 min at 4 °C. Serum was separated, aliquoted, and stored at −80 °C until further analysis. All the blood samples were collected before the results of the OGTT.

### 2.2 Serum protein extraction and tryptic digestion

To reduce interference from high-abundance serum proteins, a magnetic bead-based enrichment strategy for low-abundance proteins was used during sample pretreatment. Briefly, 30 μL of thoroughly mixed magnetic bead suspension (EVLiXiR, China) was added to 50 μL of serum sample and vortexed thoroughly. The mixture was then placed on a magnetic rack and incubated at 37 °C with shaking at 900 rpm for 2 h, after which the supernatant was removed. To further eliminate high-abundance proteins, the beads were washed twice using wash buffer.

Proteins bound to the magnetic beads were denatured using lysis buffer containing 8 M urea and 1 M Tris–HCl (pH 8.0). Disulfide bonds were reduced with 25 mM dithiothreitol (DTT) at 37 °C for 2 h. After cooling, cysteine residues were alkylated with 75 mM iodoacetamide (IAA) at room temperature in the dark for 30 min. Following alkylation, the supernatant was removed, and 25 mM ammonium bicarbonate was added to adjust the sample to conditions suitable for enzymatic digestion. Trypsin was then added at an enzyme-to-substrate ratio of 1:50 (w/w), and digestion was performed at 37 °C for 16 h. The resulting peptide solution was desalted using desalting columns, vacuum-dried at 4 °C, and finally reconstituted in a solution containing 98% water, 2% acetonitrile, and 0.1% formic acid for subsequent mass spectrometric analysis.

### 2.3 Untargeted proteomics data acquisition and protein identification

Untargeted proteomic profiling was performed using a Thermo Scientific EASY-LC 1200 nano-LC system coupled to an Orbitrap Eclipse mass spectrometer. Chromatographic separation was achieved using a binary mobile phase system, in which mobile phase A consisted of 0.1% formic acid in water and mobile phase B consisted of 0.1% formic acid in acetonitrile/water (80:20, v/v). Peptides were separated over a 60-min linear gradient from 5 to 100% mobile phase B at a constant flow rate of 300 nL/min.

Mass spectrometric data were acquired in data-independent acquisition (DIA) mode. Each acquisition cycle consisted of one full MS scan followed by one DIA fragmentation scan. Full scans were acquired in the Orbitrap analyzer at a resolution of 60,000 (at m/z 200) over an m/z range of 400 ~ 1,000. For DIA, 60 consecutive isolation windows were set across the m/z range of 400 ~ 1,000, each with a width of 10 m/z and a 1 m/z overlap between adjacent windows, to achieve comprehensive and unbiased precursor ion coverage. All ions within each isolation window were fragmented using higher-energy collisional dissociation (HCD), and fragment ions were detected in the Orbitrap at a resolution of 30,000 over an m/z range of 145 ~ 1,450. The maximum injection time was set at 54 ms to improve signal capture for low-abundance peptides. All MS/MS spectra were acquired in profile mode for quantitative accuracy and stored in centroid mode.

Instrument stability and analytical reproducibility were monitored by periodically injecting pooled quality control (QC) samples, which were prepared by mixing equal aliquots of all serum samples. The raw mass spectrometry data were processed and searched against the UniProt human proteome database using Spectronaut software. Subsequently, the processed datasets were deposited in the Figshare database (doi: 10.6084/m9.figshare.32843702).

### 2.4 Proteomics data preprocessing, differential protein screening, and bioinformatics analysis

Protein quantification data exported from Spectronaut were further processed using the R package StatTarget for QC-based correction and normalization. Although this preprocessing step did not fit a predictive model, the correction/normalization procedure incorporated GDM group information and was completed before the cross-validation analysis. Therefore, the downstream nested LASSO procedure protects against leakage from feature selection and model tuning, but it cannot completely exclude optimism related to outcome-informed upstream preprocessing. Principal component analysis (PCA) and orthogonal partial least-squares discriminant analysis (OPLS-DA) were performed using SIMCA 14.1 software. Model stability was evaluated by 200 permutation tests; an intercept of the Q^2^ regression line below zero, together with the absence of overlap between the R^2^ and Q^2^ curves, was considered indicative of no apparent overfitting.

Differential protein expression was analyzed using limma, with GDM status as the main variable and matched pair ID included as a blocking factor or fixed effect where appropriate. Based on the resulting differential protein set, Gene Ontology (GO) enrichment analysis, Kyoto Encyclopedia of Genes and Genomes (KEGG) pathway analysis, and protein–protein interaction (PPI) network construction were performed using the STRING database. Upregulated and downregulated proteins in the GDM and control groups were further subjected to separate functional enrichment analyses. All bioinformatics results were visualized using Cytoscape (v3.10.0) and the ChiPlot online platform.

### 2.5 Identification of key proteins and proteomics-integrated analysis

Based on the PPI network, the CytoHubba plug-in in Cytoscape was used to identify the top 10 hub proteins ranked by the Matthews correlation coefficient (MCC) algorithm, which were considered candidate hub proteins for further exploratory analysis. Receiver operating characteristic (ROC) curves were generated using the R package pROC (version 1.18.5), and the area under the curve (AUC) was calculated to evaluate their discriminatory performance.

For the downstream integrative analyses of dietary patterns and proteomics, candidate proteins with complete expression data and biological relevance, including APOL1, SAA4, ORM2, APOM, APOE, and TTR, were retained. Protein expression values had been preprocessed and log2-transformed, and were further standardized by Z-score transformation before association analyses.

### 2.6 Dietary assessment, food grouping, and derivation of dietary patterns

Dietary intake during pregnancy was assessed using a validated food frequency questionnaire (FFQ) specifically developed and validated for Miao pregnant women. The FFQ was administered on the same day as blood sampling at each scheduled antenatal visit. Assessments were conducted at 10–12 gestational weeks (first trimester) and 24–28 gestational weeks (second trimester), with the FFQ referring to dietary intake during the preceding trimester or the corresponding gestational period. Based on nutritional characteristics and actual intake profiles, individual food items were aggregated into predefined food groups, including Refined Grains, Legumes and Soy Products, Whole Grains, Solanaceous Vegetables, Root Vegetables, Leafy Vegetables, Cruciferous and Other Vegetables, Fermented Vegetables, Edible Fungi and Algae, Fruits, Dairy Products, Dairy Desserts, Red Meat, Poultry, Organ Meats, Processed Meats, Fish and Seafood, Eggs, Sour Soup, Beverages, Alcoholic Beverages, Nuts, Snacks, and Convenience Foods. Total energy intake was also calculated from the FFQ data. We used the same cohort of pregnant women’s information for this study (17).

Dietary patterns were derived using principal component analysis (PCA) based on dietary intake data from the full cohort of 705 pregnant women, from which the present matched case–control sample was selected. Prior to PCA, intakes of each food group were standardized. The suitability of the data for PCA was confirmed by the Kaiser–Meyer–Olkin measure of sampling adequacy (KMO = 0.783) and Bartlett’s test of sphericity (*p* < 0.001). The number of retained components was determined according to eigenvalues, scree plot inspection, and dietary interpretability. Varimax rotation was applied to improve pattern interpretation. Factor loadings were used to characterize the composition of each dietary pattern.

For the 78 participants included in the proteomics-integrated analysis, dietary pattern scores were calculated by applying the factor loading matrix derived from the full cohort to their corresponding standardized food group intakes. These pattern scores were subsequently standardized using Z-score transformation to facilitate interpretation of effect estimates in the association analyses.

### 2.7 Statistical analysis

Given the 1:1 matched case–control design, conditional logistic regression (clogit) was used to evaluate the associations between dietary pattern scores and GDM risk, with matched pair ID specified as the stratification variable. Dietary pattern scores were Z-standardized and modeled as continuous variables. Four sequential models were fitted: crude, energy-adjusted, fully adjusted (gravidity and parity), and mutually adjusted models. Age, pre-pregnancy BMI, and gestational age were controlled through the 1:1 matching design and matched-pair stratification, whereas total energy intake, gravidity, and parity were included as covariates. Physical activity was not routinely collected and therefore could not be adjusted for; education, occupation, and monthly household income were collected and are presented descriptively in Table 1 but were not included in the primary models because of the limited number of matched pairs.

**Table 1:** Baseline characteristics of the study participants (*n* = 78).

Characteristic	Category	n	%
Age at enrollment (years)	18–24	10	12.8
	25–29	16	20.5
	30–35	28	35.9
	≥35	24	30.8
Pre-pregnancy BMI (kg/m^2^)	<18.5	4	5.1
	18.5–23.9	43	55.1
	≥24	31	39.7
Education (years)	< 9	41	52.6
	9–12	12	15.4
	≥13	25	32.1
Occupation	Employed	38	48.7
	Unemployed	40	51.3
Monthly household income (CNY)	1,000 - 3,000	39	50
	≥ 3,000	39	50
Gravidity	1	32	41.0
	≥2	46	59.0
Parity	Nulliparous	24	30.8
	Multiparous	54	69.2
Gestational age at sampling/FFQ (weeks)*	First trimester	24	12.00 (10.00–12.00)
	Second trimester	54	26.00 (25.00–27.00)

BMI, body mass index; CNY, Chinese Yuan.

*Continuous variables with skewed distributions are presented as median (interquartile range, IQR).

Differential protein screening was performed using the limma package. Both nominal *p* values and Benjamini–Hochberg false discovery rate (FDR)-adjusted q values were calculated. Using the exploratory criteria of |log2FC| > log2(1.2) and nominal *p* ≤ 0.05, 34 candidate differentially expressed proteins were identified for descriptive pathway and network analyses; these results were not used as a pre-filter for the nested LASSO analysis. To address data leakage from outcome-informed feature selection, all 344 proteins that passed the predefined quality-control criteria were entered into a leave-one-matched-pair-out nested LASSO feature-selection procedure without prior differential-expression filtering. In each outer iteration, one complete matched case–control pair was held out as the test set, while LASSO penalized logistic regression and inner leave-one-out cross-validation were conducted exclusively within the remaining training pairs to select the penalty parameter using the 1-SE rule. The resulting model was then applied to the held-out pair, and predictions from all outer iterations were pooled to estimate internal discrimination. The 95% CI of the primary nested-CV AUC was estimated by bootstrap resampling of the 39 matched pairs, preserving the original matched structure. Selection frequencies across the 39 outer iterations were summarized descriptively. Because the upstream QC correction/normalization was completed before cross-validation and incorporated GDM group information, the nested procedure cannot be regarded as completely independent of all upstream preprocessing. A 1,000-repetition matched-pair label permutation analysis was additionally performed as an empirical robustness check; labels were exchanged or retained within each matched pair and the downstream 344-protein nested LASSO procedure was repeated. Associations between dietary pattern scores and candidate proteins were evaluated using linear mixed-effects models with matched pair ID included as a random intercept. APOM and SAA4 were selected to construct a Z-standardized core protein score, and their joint associations with dietary pattern scores and GDM risk were assessed using conditional logistic regression adjusted for total energy intake, gravidity, and parity. Bootstrap internal validation was additionally performed. All analyses were conducted in R (version 4.3.2), and two-sided *p* < 0.05 was considered statistically significant.

## 3 Results

### 3.1 Baseline characteristics

As shown in Table 1, 78 pregnant women were included in the present study. The largest proportion of participants were aged 30–35 years (35.9%), followed by those aged ≥35 years (30.8%), indicating that a considerable proportion of the study population was aged 30 years or older. More than half of the participants had a normal pre-pregnancy BMI (18.5–23.9 kg/m^2^; 55.1%), whereas 39.7% were overweight or obese and 5.1% were underweight. Regarding sociodemographic characteristics, 52.6% had ≤9 years of education, and employment status was nearly evenly distributed between employed and unemployed participants. Monthly household income was equally distributed between the 1,000–3,000 CNY and ≥3,000 CNY groups. With respect to reproductive history, 59.0% of participants had two or more pregnancies, and 69.2% were multiparous. These findings indicate that the study sample was mainly composed of women with normal pre-pregnancy BMI, a relatively high proportion of participants aged 30 years or older, and a high proportion of multiparous women. Regarding gestational age at sampling and FFQ administration, 24 participants were assessed during the first trimester at a median gestational age of 12 weeks (IQR, 10–12), whereas 54 participants were assessed during the second trimester at a median gestational age of 26 weeks (IQR, 25–27).

### 3.2 Composition characteristics of the derived dietary patterns

Given the relatively limited sample size of the proteomics subset (*n* = 78), dietary patterns were derived from a previously established cohort of 705 pregnant women to enhance the stability and representativeness of pattern extraction. Specifically, principal component analysis (PCA) was conducted on dietary intake data from the larger cohort, and the resulting pattern structure was subsequently applied to the 78 participants included in the present study for further analysis of dietary pattern–protein associations. Following varimax rotation, two major dietary patterns were identified: the Processed Dietary Pattern and the Balanced Dietary Pattern (Table 2).

**Table 2:** Factor loading matrix of dietary patterns derived from principal component analysis with varimax rotation.

Food group	Balanced dietary pattern	Processed dietary pattern
Refined grains	---	0.36
Legumes and soy products	---	---
Whole grains	0.5	---
Solanaceous vegetables	0.402	---
Root vegetables	0.499	---
Leafy vegetables	0.361	---
Cruciferous and other vegetables	0.43	---
Fermented vegetables	---	---
Edible fungi and algae	0.547	---
Fruits	0.396	---
Dairy products	0.435	−0.421
Dairy desserts	---	0.439
Red meat	---	0.328
Poultry	0.378	---
Organ meats	0.353	---
Processed meats	---	0.395
Fish and seafood	0.501	---
Eggs	0.471	−0.372
Sour soup	---	---
Beverages	---	0.537
Alcoholic beverages	---	0.454
Nuts	---	---
Snacks	---	0.311
Convenience foods	---	0.354
Explained variance (%)	11.54	8.4

Values represent factor loadings (Pearson correlation coefficients between each food group and the corresponding dietary pattern score) derived from principal component analysis. Only factor loadings with an absolute value ≥0.3 are displayed; remaining entries are denoted ---. Food groups are ordered as presented in the original questionnaire.

The Processed Dietary Pattern was characterized by high factor loadings for beverages (0.537), alcoholic beverages (0.454), dairy desserts (0.439), processed meat (0.395), refined grains (0.360), and convenience foods (0.354), with additional contributions from red meat (0.328) and snacks (0.311). This pattern reflected a dietary structure dominated by ultra-processed foods, sweetened products, beverages and alcohol, and processed meat. In contrast, the Balanced Dietary Pattern was principally defined by high loadings for edible fungi and algae (0.547), fish and seafood (0.501), whole grains (0.500), root vegetables (0.499), eggs (0.471), dairy products (0.435), cruciferous and other vegetables (0.430), solanaceous vegetables (0.402), and fruits (0.396), with additional contributions from leafy vegetables (0.361), poultry (0.378), and organ meats (0.353). This pattern represented a nutritionally diverse dietary structure characterized by higher intakes of whole grains, vegetables, fruits, fish and seafood, eggs, and dairy products.

### 3.3 Associations between dietary pattern scores and GDM

Following identification of the Processed and Balanced Dietary Patterns, conditional logistic regression was used to evaluate their associations with GDM risk across four progressively adjusted models (Table 3). The Processed Dietary Pattern was positively associated with GDM risk throughout: the association was significant in the crude model, attenuated to borderline significance after adjustment for total energy intake, and significant again in both the fully adjusted and mutually adjusted models, with the strongest association observed after mutual adjustment for the Balanced Dietary Pattern.

**Table 3:** Associations between dietary pattern scores and the risk of GDM.

Exposure	Crude model		Energy-adjusted model		Fully adjusted model		Mutually adjusted model	
	OR (95%CI)	*P*	OR (95%CI)	*P*	OR (95%CI)	*P*	OR (95%CI)	*P*
Processed Dietary	2.14 (1.12, 4.10)	0.021	2.06 (0.97, 4.38)	0.061	2.33 (1.08, 5.04)	0.031	2.55 (1.25, 5.23)	0.010
Balanced Dietary	0.95 (0.60, 1.51)	0.84	0.67 (0.37, 1.23)	0.198	0.57 (0.28, 1.15)	0.116	0.98 (0.55, 1.75)	0.949

OR, odds ratio; CI, confidence interval.

Each exposure unit represents a 1-SD increment in the Z-standardized dietary pattern score.

The energy-adjusted model was additionally adjusted for total energy intake relative to the crude model.

The fully adjusted model was further adjusted for total energy intake, gravidity and parity.

The mutually adjusted model additionally included both dietary pattern scores simultaneously.

The Balanced Dietary Pattern showed no significant association with GDM risk in any model. Although point estimates trended below 1 after energy and full adjustment, all confidence intervals crossed the null, and the association was further attenuated toward unity after mutual adjustment for the Processed Dietary Pattern. Taken together, the Processed Dietary Pattern showed the clearest dietary association with higher GDM risk in this matched sample, whereas the Balanced Dietary Pattern showed no statistically significant protective association in this population, suggesting that the Processed Dietary Pattern represents the more prominent dietary risk signal for GDM.

### 3.4 Serum proteomic characteristics and differential protein analysis in GDM

#### 3.4.1 Protein identification, quality control, and global proteomic profiling

A total of 1,658 proteins were initially identified by Spectronaut analysis. After excluding proteins with >30% missing values, 416 proteins were retained for downstream analyses; following quality control (QC) correction, 344 proteins met the predefined QC criteria and were included in subsequent analyses. As shown in Supplementary Figure 1A, PCA demonstrated that all QC samples clustered tightly together, confirming good instrumental stability and high data reliability throughout the detection process. Supervised orthogonal partial least squares discriminant analysis (OPLS-DA) clearly separated the GDM group from the healthy control group, indicating marked alterations in the serum proteomic profiles of pregnant women with GDM (Supplementary Figure 1B). The OPLS-DA model was assessed by 200 permutation tests as an exploratory check of model stability (Supplementary Figure 1C).

#### 3.4.2 Identification and functional characterization of differentially expressed proteins

Exploratory differential protein screening using |log2FC| > log2(1.2) and nominal *p* ≤ 0.05 identified 34 candidate differentially expressed proteins (11 upregulated and 23 downregulated; Figure 1A). FDR-adjusted q values were additionally calculated and are presented in Supplementary Table 1. Only four of the 34 candidate proteins (APOL1, PRSS1, TCEAL6, and SAA4) had q < 0.05 after Benjamini–Hochberg correction. Because of the limited sample size and high-dimensional nature of the proteomic data, these findings were considered hypothesis-generating candidates rather than definitive biomarkers. The DEPs exhibited distinct group-specific clustering (Figure 1B). Gene Ontology (GO) analysis revealed that these proteins were primarily enriched in extracellular exosomes, lipoprotein particles (HDL, VLDL, and LDL), and keratin filaments (Figure 2A). Functionally, they were predominantly involved in lipid metabolism, immune responses, and cytoskeletal reorganization. Furthermore, stage-specific analysis identified 15 first-trimester differentially expressed proteins, which were primarily characterized by dysregulation in the inflammation–complement–lipid metabolism axis (Supplementary Figure 2).

**Figure 1:**
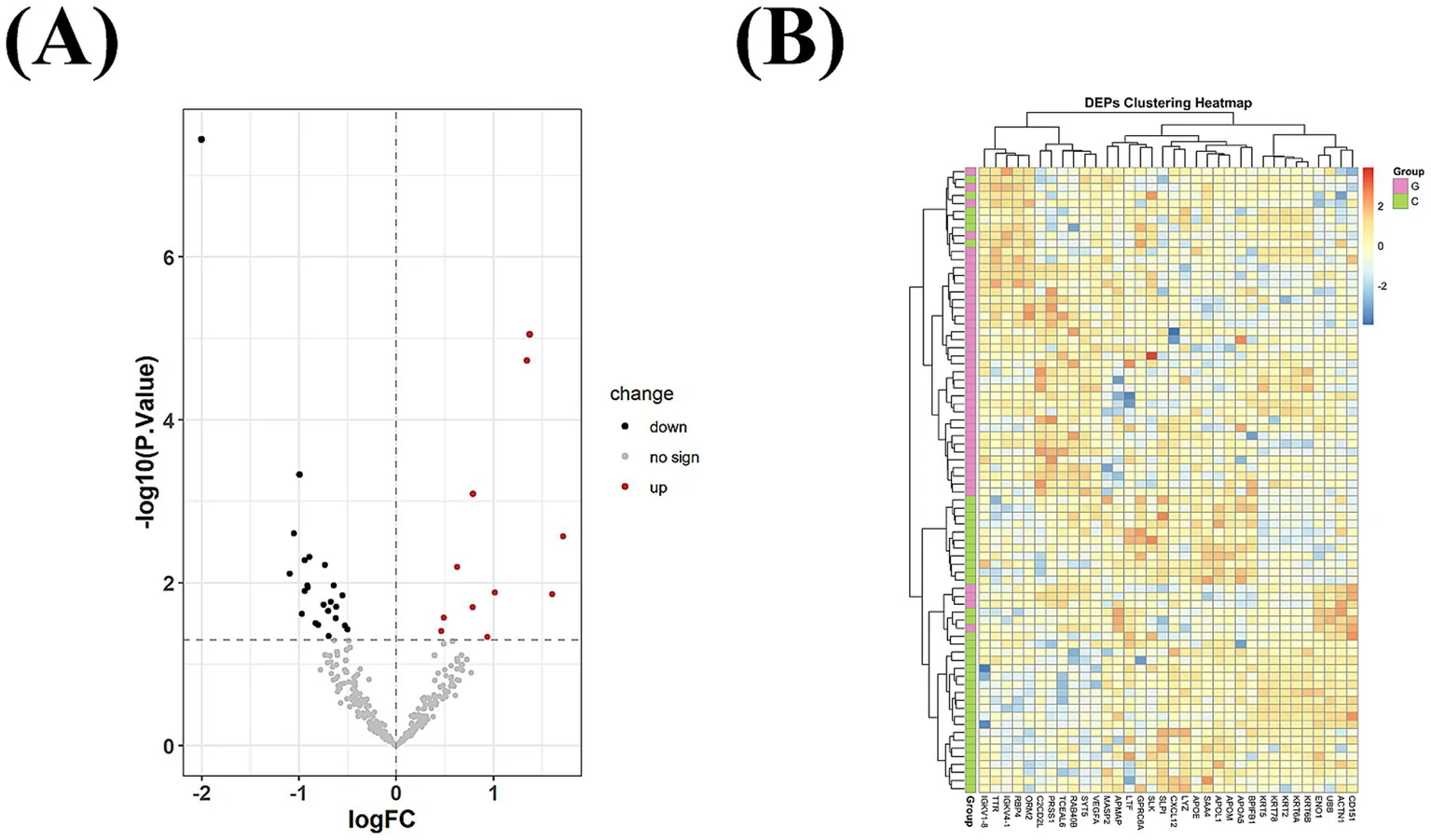
Volcano plot and hierarchical clustering heatmap of all differentially expressed proteins. **(A)** Volcano plot comparing the GDM and control groups. Red: Upregulated proteins; black: Downregulated proteins. **(B)** Heatmap of all 34 DEPs. G/C, GDM/control.

**Figure 2:**
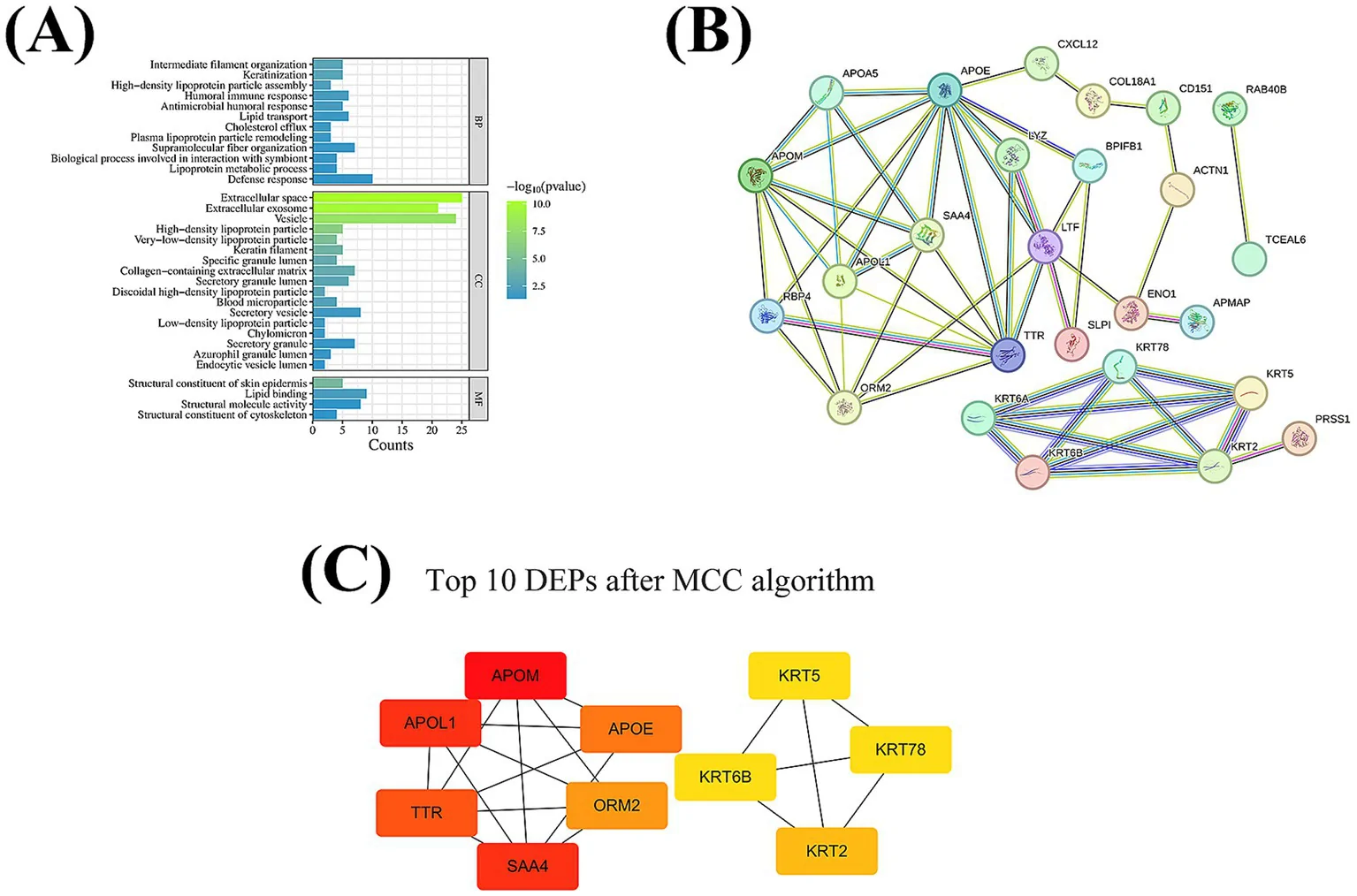
Bioinformatic and PPI network analysis of DEPs between the GDM and control groups. **(A)** GO annotations across biological process (BP), cellular component (CC), and molecular function (MF) categories; color intensity indicates –log_10_ (*p* value); bar length represents gene count. **(B)** PPI network of the 34 DEPs. **(C)** Top 10 hub proteins ranked by the MCC algorithm; color intensity corresponds to hub score.

A protein–protein interaction (PPI) network of the 34 DEPs revealed three functional modules: APOE-centered lipid metabolism, LTF/LYZ-mediated immune defense, and keratin-associated structural alterations (Figure 2B). CytoHubba MCC ranking identified the top 10 overall hub proteins, divided into lipid/inflammation-related (APOM, APOL1, APOE, SAA4, TTR, and ORM2) and structural (KRT2, KRT5, KRT6B, and KRT78) hubs (Figure 2C). Applying this analytical pipeline specifically to the 15 first-trimester DEPs (Supplementary Figure 2A), we identified seven early-stage hub proteins (C8G, APOL1, MASP2, SAA4, KRT6B, KRT5, and KRT16; Supplementary Figure 2B). Exploratory ROC analysis showed discriminatory signals among these first-trimester hub proteins, with SAA4 showing the highest AUC (0.965) (Supplementary Figure 2C). These findings were not cross-validated and were considered exploratory.

### 3.5 Adjusted associations of dietary pattern scores with candidate proteins

To further explore the potential biological links between dietary patterns and GDM-related proteins, linear mixed-effects models were used to evaluate the associations between the two dietary pattern scores and candidate protein expression levels (Table 4). After adjustment for total energy intake, gravidity, and parity, the processed dietary pattern score was inversely associated with APOM expression (*β* = −0.308, 95% CI: −0.595 to −0.020, *p* = 0.036), and showed a borderline inverse association with SAA4 (*β* = −0.285, 95% CI: −0.573 to 0.003, *p* = 0.052). In addition, the processed dietary pattern scores also showed inverse trends with APOL1 and APOE, although these associations were not statistically significant. By contrast, positive but non-significant trends were observed for ORM2 and TTR.

**Table 4:** Associations of dietary pattern scores with candidate proteins.

Protein	Processed *β* (95%CI)	*P*	Balanced *β* (95%CI)	*P*
APOL1	−0.188 (−0.483, 0.106)	0.207	0.029 (−0.247, 0.305)	0.835
SAA4	−0.285 (−0.573, 0.003)	0.052	0.079 (−0.195, 0.352)	0.567
ORM2	0.082 (−0.212, 0.376)	0.581	−0.095 (−0.366, 0.177)	0.49
APOM	−0.308 (−0.595, −0.020)	0.036	0.155 (−0.117, 0.427)	0.259
APOE	−0.175 (−0.470, 0.120)	0.241	−0.005 (−0.281, 0.272)	0.974
TTR	0.122 (−0.164, 0.408)	0.397	−0.083 (−0.348, 0.183)	0.538

Processed represents Processed dietary pattern; Balanced represents Balanced dietary pattern.

APOL1, Apolipoprotein L1; SAA4, Serum amyloid A-4 protein; ORM2, Alpha-1-acid glycoprotein 2; APOM, Apolipoprotein M; APOE, Apolipoprotein E; TTR, Transthyretin.

The β and its 95% Cl were estimated by a linear mixed model, with the number of matched groups set at 1:1 and included as a random intercept in the model.

The adjustment variables included energy, gravidity and parity.

*p* = 0.052 was considered borderline evidence and was not interpreted as statistically significant.

In contrast, the balanced dietary pattern score was not significantly associated with any of the candidate proteins. Although weak positive trends were observed for APOM and SAA4, and weak negative trends for ORM2, TTR, and APOE, all 95% confidence intervals crossed zero. These results indicate that APOM showed the most consistent diet-related association, whereas SAA4 provided weaker, borderline evidence in the same direction.

### 3.6 Joint associations of dietary pattern scores and core protein score with GDM risk

We next performed conditional logistic regression analyses within the 1:1 matched case–control framework to evaluate the joint associations of dietary pattern scores and the APOM/SAA4 core protein score with GDM status (Table 5). The core protein score was constructed from APOM and SAA4 for exploratory integrative analysis.

**Table 5:** Stepwise multivariable conditional logistic regression analyses for the joint associations of dietary pattern scores and core protein score with GDM risk.

Dietary pattern	Model	Dietary pattern score OR (95% CI)	*P*	Core protein score OR (95% CI)	*P*
Processed dietary pattern	Crude	2.47 (1.03, 5.94)	0.043	5.72 (1.71, 19.18)	0.005
	Basic adjusted	2.02 (0.79, 5.17)	0.143	7.74 (1.84, 32.64)	0.005
	Further adjusted	1.80 (0.67, 4.89)	0.246	8.30 (1.87, 36.82)	0.005
Balanced dietary pattern	Crude	1.13 (0.61, 2.08)	0.697	5.17 (1.79, 14.93)	0.002
	Basic adjusted	0.71 (0.31, 1.64)	0.423	7.08 (1.85, 27.04)	0.004
	Further adjusted	0.50 (0.18, 1.44)	0.201	8.99 (1.96, 41.34)	0.005

All models were fitted using conditional logistic regression stratified by matched-pair ID. Crude Model was unadjusted; Basic adjusted Model was additionally adjusted for total energy intake; Further adjusted Model was further adjusted for gravidity and parity.

Age, pre-pregnancy BMI, and gestational age were controlled by the 1:1 matching design and matched-pair stratification.

The core protein score was calculated as the mean of risk-direction-coded Z scores of APOM and SAA4: core protein score = [−Z(APOM) − Z(SAA4)]/2.

In the processed dietary pattern joint models, the core protein score remained associated with GDM across all models. In the crude model, the OR for the core protein score was 5.72 (95% CI: 1.71–19.18, *p* = 0.005); after adjustment for total energy intake, the OR increased to 7.74 (95% CI: 1.84–32.64, *p* = 0.005); after further adjustment for gravidity and parity, the association remained significant (OR = 8.30, 95% CI: 1.87–36.82, *p* = 0.005). In contrast, the processed dietary pattern score itself was significantly associated with GDM only in the crude model (OR = 2.47, 95% CI: 1.03–5.94, *p* = 0.043), but was no longer significant after further adjustment.

Similarly, in the balanced dietary pattern joint models, the core protein score also showed stable and significant associations with GDM, with ORs of 5.17 (95% CI: 1.79–14.93, *p* = 0.002), 7.08 (95% CI: 1.85–27.04, *p* = 0.004), and 8.99 (95% CI: 1.96–41.34, *p* = 0.005) in the crude, energy-adjusted, and further adjusted models, respectively. By contrast, the balanced dietary pattern score was not significantly associated with GDM in any of the models.

Taken together, after inclusion of the core protein score, the associations between dietary pattern scores and GDM were attenuated, whereas the core protein score remained associated with GDM across the sequential matched models. These findings should be interpreted as exploratory joint associations rather than evidence that the proteomic measures are causally more proximal to GDM risk.

### 3.7 Cross-validated feature selection and exploratory integrative analysis

To address data leakage from prior differential-expression screening, a leave-one-matched-pair-out nested LASSO feature-selection procedure was performed using all 344 proteins that passed quality-control criteria. In each outer iteration, one complete matched case–control pair was held out as the test set, while LASSO penalized logistic regression and inner leave-one-out cross-validation were conducted exclusively within the remaining training pairs to select the penalty parameter using the 1-SE rule. The resulting model was then applied to the held-out pair, and predictions from all outer iterations were pooled to estimate internal discrimination. The fully nested leave-one-matched-pair-out procedure yielded an AUC of 0.953 (95% CI: 0.894–0.992; Supplementary Table 3). Sensitivity analyses yielded AUCs of 0.916 after removal of common contaminant/technical-related proteins and 0.918 after further removal of immunoglobulin variable-region proteins (Supplementary Table 5). Because the upstream QC correction/normalization had been completed before cross-validation and incorporated GDM group information, this estimate should not be interpreted as completely free of preprocessing-related optimism. It is therefore presented as exploratory internal discrimination rather than externally validated predictive performance.

Across the 39 outer iterations, 35 proteins received non-zero LASSO coefficients in at least one iteration, whereas the remaining 309 of the 344 proteins were never selected (Supplementary Table 2). Six proteins (APOL1, GPRC6A, PRSS1, RFC1, SAA4, and TCEAL6) were selected in all 39 iterations (100%). ACTN1 and LGALS3BP were selected in 37 iterations (94.9%), PCYOX1 in 32 iterations (82.1%), and TGFBI in 30 iterations (76.9%). Overall, selection frequencies varied substantially across proteins, indicating that feature stability remained sensitive to resampling in this small cohort.

APOM was not selected in any of the 39 outer iterations in the 344-protein nested LASSO analysis. Nevertheless, APOM was retained for the subsequent exploratory integrative analysis for a distinct, hypothesis-driven reason: it was identified as a PPI hub and showed the most consistent inverse association with the processed dietary pattern among the candidate proteins. Thus, the data-driven nested LASSO and the biologically motivated APOM/SAA4 integrative analysis served different purposes. APOM’s lack of recurrence in the nested LASSO analysis was explicitly acknowledged, and its inclusion should not be interpreted as evidence of stable data-driven feature selection.

As an empirical robustness check, 1,000 matched-pair label permutations were performed, with the GDM/control labels randomly exchanged or retained within each pair and the downstream 344-protein nested LASSO procedure repeated. No permuted AUC reached the observed AUC of 0.953, corresponding to an empirical *p* = 0.001. Because the upstream QC correction/normalization was performed before permutation and incorporated GDM group information, this permutation result was interpreted as a conditional empirical check of the downstream modeling procedure rather than as evidence that the entire analytical pipeline was free of information leakage. These sensitivity analyses provided additional evidence that the observed internal discrimination was not attributable solely to contaminant/technical-related proteins or immunoglobulin variable-region proteins (Figure 3, Table 6).

**Figure 3:**
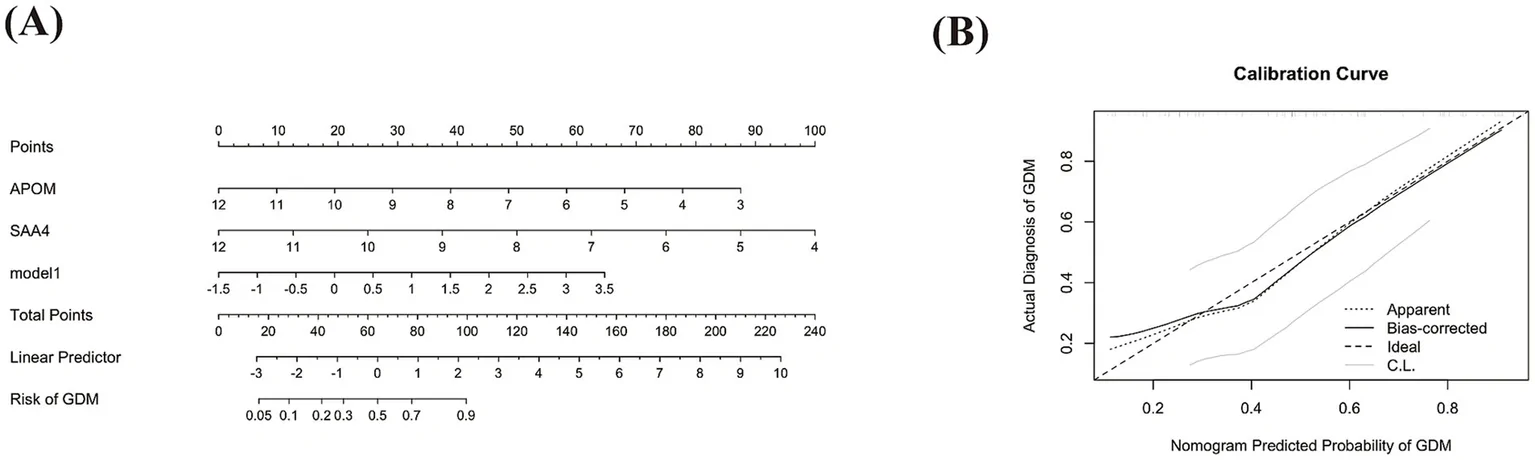
Exploratory proof-of-concept nomogram illustrating the joint associations of APOM, SAA4, and the processed dietary pattern with GDM status. **(A)** The exploratory nomogram illustrating the joint associations of APOM, SAA4, and the processed dietary pattern (model 1). The total points are calculated by summing the points assigned to each predictor and mapped to the model-derived probability scale. **(B)** Calibration curve evaluated using 1,000 bootstrap resamples. This nomogram is intended solely as an exploratory proof-of-concept illustration and should not be interpreted as a clinically applicable prediction model.

**Table 6:** Exploratory multivariable associations of APOM, SAA4, and the processed dietary pattern with GDM status.

Variables	Model 1 OR (95% CI)	*p* value	Model 2 OR (95% CI)	*P* value
APOM concentration	0.63 (0.22–1.74)	0.370	0.55 (0.19–1.59)	0.268
SAA4 concentration	0.46 (0.20–1.01)	0.053	0.46 (0.21–1.04)	0.062
Processed dietary pattern	-	-	2.22 (1.14–4.32)	0.019
Model evaluation metrics				
Likelihood Ratio	17.48	<0.001	24.42	<0.001
Nagelkerke R^2^	0.268	-	0.358	-
C-index (Apparent AUC)	0.755	-	0.801	-
Bootstrap-corrected C-index (AUC)	0.739	-	0.777	-
Brier score	0.2	-	0.182	-

OR is the odds ratio derived from unconditional logistic regression models; 95% CI is the 95% confidence interval.

Model 1 includes only APOM and SAA4; Model 2 incorporates both proteins and the processed dietary pattern.

The C-index values shown here represent apparent model discrimination within the derivation cohort; bootstrap correction provides an internal optimism-adjusted estimate. These estimates should not be interpreted as externally validated predictive performance.

## 4 Discussion

### 4.1 Principal findings

In this matched case–control study of Miao pregnant women, dietary pattern analysis was integrated with serum proteomics to interrogate the diet–protein–GDM relationship. The principal findings were as follows: the processed dietary pattern was associated with GDM status; the serum proteomic profile showed alterations involving lipid/lipoprotein remodeling, immune-inflammatory responses, and structural pathways; nested leave-one-pair-out LASSO analysis across all 344 quality-controlled proteins yielded heterogeneous feature-selection frequencies, with six proteins selected in all 39 outer iterations; and the processed dietary pattern was inversely associated with APOM and showed a borderline inverse association with SAA4. APOM was subsequently retained for exploratory integration primarily because of its dietary association, PPI-hub status, and biological relevance. The resulting APOM/SAA4–diet model was considered a proof-of-concept analysis rather than a clinical prediction model.

### 4.2 Processed dietary pattern as the dominant dietary risk signal for GDM

The positive association observed between the processed dietary pattern and GDM risk is epidemiologically coherent. Recent systematic reviews and meta-analyses consistently show that Western or processed dietary patterns are associated with higher GDM risk (31, 32). In a 2024 dose–response meta-analysis, the highest category of UPF intake was associated with a 19% increased risk of GDM (pooled RR = 1.19), and notably, every 100 g/day increment in UPF intake was associated with a 27% increase in GDM risk (RR = 1.27) (33). This pattern is biologically plausible. Processed dietary patterns are typically enriched in refined grains, processed meats, sweetened beverages, snacks, and energy-dense foods, all of which can promote insulin resistance, hepatic lipid dysregulation, and chronic low-grade inflammation (34, 35). Supporting this mechanistic backdrop, a 2024 large-scale proteomic study showed that a higher dietary inflammatory index was associated with broad shifts in circulating inflammatory proteins, consistent with the notion that unhealthy dietary exposure leaves a measurable inflammatory-proteomic signature (36). Within this context, the present findings suggest that the processed dietary pattern was associated with alterations in disease-relevant circulating proteins in the present cohort, although the temporal ordering of these associations cannot be established.

### 4.3 Limited detectability of a protective association for the balanced dietary pattern

The absence of a statistically significant protective association for the balanced dietary pattern should not be interpreted as evidence of no biological benefit. Rather, it likely reflects a combination of modest effect size, limited exposure contrast, and restricted statistical power. Protective dietary patterns often exert weaker and more diffuse effects than strongly adverse dietary patterns, making them more difficult to detect in a 39-pair matched design. Moreover, a balanced pattern is inherently heterogeneous, incorporating several food groups with potentially different metabolic implications, which may dilute a single dominant protective signal. This interpretation is also compatible with the broader literature, in which healthier dietary patterns generally trend toward lower GDM risk but often show substantial between-study heterogeneity, ethnic background, habitual dietary structure, and food substitution patterns may materially influence the detectability of protective associations (37). In the present population, the contrast in processed food consumption may have been larger and more discriminating than the contrast in adherence to healthier eating, making the processed dietary pattern a more detectable risk phenotype than the balanced dietary pattern as a protective one.

### 4.4 The serum proteomic landscape of GDM: coordinated disturbance in lipid metabolism, immune inflammation, and structural adaptation

The proteomic results indicate that GDM-related molecular differences involved coordinated patterns across lipid/lipoprotein remodeling, immune-inflammatory and host-defense responses, and cytoskeletal or structural pathways. These patterns are better interpreted as a composite proteomic profile associated with GDM status rather than as evidence of a single causal process.

The lipid-related module was the most prominent. A cluster centered on APOE and linked to APOM, APOL1, SAA4, APOA5, and APMAP pointed to disturbed HDL-related transport and maternal lipid remodeling (38, 39). This specific apolipoprotein cluster and its behavior in inflammatory and metabolic disease states is thoroughly documented in modern HDL proteomics (38, 40). Normal pregnancy involves a highly regulated physiological lipid adaptation, transitioning from an anabolic state to a catabolic state to ensure adequate nutrient transfer to the fetus, and this process is amplified and exacerbated toward profound dysregulation in GDM (41, 42). In this context, the inverse associations of the processed dietary pattern with APOM and SAA4 may be consistent with alterations in an HDL-associated proteomic network.

The immune-inflammatory module adds a second layer of interpretation. The enrichment of proteins such as LTF, LYZ, and complement-related components suggests that GDM is accompanied by immunometabolic reprogramming rather than by a purely metabolic abnormality (43, 44). These signals are better interpreted as evidence of altered immune homeostasis and host-defense activity than as isolated inflammatory events (27). Their co-occurrence with lipid-related proteins also suggests that inflammatory activation and lipid dysregulation are biologically linked processes within the GDM proteomic profile (45). The structural module, dominated by keratin family proteins, points to cytoskeletal and epithelial remodeling, their clustering implies that placental or pregnancy-related barrier adaptation may accompany metabolic and inflammatory stress (27, 46). This finding may broaden the interpretation of GDM from a disorder of glucose regulation alone to a condition involving simultaneous metabolic, immune, and tissue-adaptive responses.

Trimester-stratified analyses were conducted to explore early-pregnancy proteomic differences, with a specific focus on the first trimester. First-trimester changes were prominently concentrated along an inflammation-complement-lipid metabolism axis, these findings serve primarily as hypothesis-generating observations requiring validation in larger prospective cohorts (47).

### 4.5 Diet-related proteins as molecular bridges: focus on APOM and SAA4

Among the candidate proteins, APOM and SAA4 showed the most consistent inverse associations with the processed dietary pattern, although the evidence for SAA4 was weaker. APOM is an HDL-associated apolipoprotein and a major carrier of sphingosine-1-phosphate (S1P), and the APOM/S1P axis has been implicated in inflammation, cholesterol metabolism, endothelial barrier integrity, triglyceride turnover, and metabolic regulation (48, 49). In the present study, the processed dietary pattern was inversely associated with APOM, supporting an association between dietary pattern characteristics and alterations in HDL-related proteomic profiles. The interpretation of SAA4 requires greater caution. Unlike SAA1 and SAA2, which are inducible acute-phase proteins, SAA4 is constitutively expressed and is predominantly associated with HDL particles. Therefore, SAA4 should not be interpreted as a direct surrogate of the classical acute-phase inflammatory response. One possible explanation is that the observed inverse association may reflect changes in HDL particle composition, remodeling, or the distribution of SAA4 among circulating lipoprotein particles rather than a simple change in systemic inflammation. Previous studies have shown that the interaction between SAA proteins and HDL can alter HDL composition and function, providing a biologically plausible context for diet-related changes in circulating SAA4 (50–52).

However, this interpretation remains speculative. The present study did not directly assess HDL subclasses, HDL lipid composition, SAA4 partitioning across lipoproteins, or genetic determinants of SAA4 expression. Moreover, the inverse association between the processed dietary pattern and SAA4 was borderline (*β* = −0.285, *p* = 0.052), and the study included only 78 participants. Thus, a chance finding cannot be excluded, and the observed association should be regarded as hypothesis-generating rather than evidence of a protective effect. Replication in larger studies with detailed HDL characterization and genetic or mechanistic measurements will be necessary to determine whether this observation reflects a reproducible biological phenomenon.

### 4.6 From diet to disease: interpreting the multivariable joint model

The present study is best interpreted as exploratory evidence across complementary analytical layers rather than as confirmation of a single biomarker pathway. The processed dietary pattern was associated with higher GDM risk; it was also inversely related to APOM and, to a lesser extent, SAA4; and the core protein score remained associated with GDM after inclusion in matched models. The nested LASSO analysis was designed to reduce leakage from differential-expression-based feature selection and model tuning. Its internal discrimination estimate should nevertheless be interpreted cautiously because upstream QC correction/normalization incorporated GDM group information before cross-validation. APOM was not treated as a stable data-driven LASSO feature; instead, it was retained for integration based on complementary PPI and diet–protein evidence. These findings therefore support APOM and SAA4 as exploratory candidates for further investigation rather than validated biomarkers.

This interpretation is consistent with the broader direction of contemporary diet–proteome research. In 2023, ARIC/FHS analyses showed that healthy dietary patterns correspond to specific circulating protein profiles, and another journal reported that proteomic signatures reflecting adherence and biological response to healthy dietary patterns were prospectively associated with lower risks of diabetes and other chronic diseases, even after accounting for the dietary patterns themselves (30). In the present study, the observed relationships among dietary pattern, proteomic features, and GDM status should be interpreted as associative, and their temporal ordering or causal direction cannot be established.

### 4.7 Limitations

Several limitations should be considered. First, the relatively small sample size (*n* = 78; 39 GDM cases and 39 controls) substantially limits statistical power for high-dimensional proteomic analyses. Among the 34 exploratory candidate DEPs, only four proteins (APOL1, PRSS1, TCEAL6, and SAA4) had q < 0.05 after Benjamini–Hochberg correction, underscoring the uncertainty associated with multiple testing. Accordingly, the identified proteins should be considered hypothesis-generating candidates rather than definitive biomarkers.

Second, the nested leave-one-pair-out analysis used all 344 quality-controlled proteins and performed feature selection and LASSO tuning within the training folds. This reduced the potential for leakage from downstream feature selection, but the upstream QC correction/normalization was completed before cross-validation and incorporated GDM group information. Accordingly, complete independence of the outer test sets from all preprocessing steps cannot be guaranteed. Internal validation cannot replace independent external validation, and the resulting AUC should be interpreted as exploratory internal discrimination only.

Third, APOM was retained for the integrative model despite its absence from the selected features of the 344-protein nested LASSO analysis because of its PPI-hub status, its statistically significant association with the processed dietary pattern, and its biological relevance. This was a hypothesis-driven exploratory prioritization and may introduce subjective feature selection.

Finally, physical activity was not routinely collected and could not be adjusted for. Although education, occupation, and monthly household income were collected and are presented descriptively in Table 1, they were not included in the primary models because of the limited number of matched pairs. Residual confounding therefore cannot be excluded. The multivariable model and nomogram were developed within the same study population and should be regarded strictly as proof-of-concept analyses rather than clinically applicable prediction models. Their reproducibility and generalizability require evaluation in larger and independent cohorts.

## 5 Conclusion

This study found that a processed dietary pattern was associated with GDM status among Miao pregnant women and with altered circulating APOM and SAA4 profiles. In exploratory high-dimensional proteomic analysis, a nested leave-one-pair-out procedure using all 344 quality-controlled proteins was used to assess feature stability while avoiding prior differential-expression filtering within the validation loop. SAA4 was selected in all 39 outer iterations, whereas selection frequencies varied across other proteins. APOM was retained for subsequent integrative analysis based on complementary PPI, diet–protein, and biological evidence despite its lower data-driven selection stability. The APOM/SAA4–diet model is considered a proof-of-concept analysis only and should not be interpreted as a clinically applicable prediction model. Because upstream QC correction/normalization incorporated GDM group information before cross-validation, the internal discrimination estimate should be interpreted cautiously. Larger, independent, and prospective studies are required to determine the reproducibility and generalizability of these findings.

## Data Availability

The datasets presented in this study can be found in online repositories. The names of the repository/repositories and accession number(s) can be found at: https://figshare.com/, 10.6084/m9.figshare.32843702.
