# Supplementary material for: A processed dietary pattern is associated with gestational diabetes mellitus and altered APOM and SAA4 proteomic signatures in Miao women
Source: Front Nutr. 2026 Oct 28;13:1933838. doi: 10.3389/fnut.2026.1933838 (PMC13752567; doi:10.3389/fnut.2026.1933838)
Supplement: Supplementary file 1 [file Supplementary_file_1.DOCX]

**Supplementary Materials**

| **Supplementary Table 1. Characteristics of differentially expressed proteins between the GDM and control groups** | | | | | | | |
| --- | --- | --- | --- | --- | --- | --- | --- |
| **No.** | **Gene** | **Protein name** | **Uniprot ID** | **log_2_FC** | ***P* value** | **q value** | **Direction** |
| 1 | APOL1 | Apolipoprotein L1 | O14791 | -2.004 | <0.001 | <0.001 | down |
| 2 | PRSS1 | Serine protease 1 | P07477 | 1.371 | <0.001 | 0.002 | up |
| 3 | TCEAL6 | Transcription elongation factor A protein-like 6 | Q6IPX3 | 1.342 | <0.001 | 0.002 | up |
| 4 | SAA4 | Serum amyloid A-4 protein | P35542 | -0.997 | <0.001 | 0.040 | down |
| 5 | C2CD2L | Phospholipid transfer protein C2CD2L | O14523 | 0.785 | <0.001 | 0.055 | up |
| 6 | MASP2 | Mannan-binding lectin serine protease 2 | O00187 | -1.052 | 0.0024 | 0.133 | down |
| 7 | ORM2 | Alpha-1-acid glycoprotein 2 | P19652 | 1.718 | 0.003 | 0.133 | up |
| 8 | UBB | Polyubiquitin-B | P0CG47 | -0.891 | 0.005 | 0.200 | down |
| 9 | BPIFB1 | BPI fold-containing family B member 1 | Q8TDL5 | -0.939 | 0.005 | 0.200 | down |
| 10 | APOM | Apolipoprotein M | O95445 | -0.731 | 0.006 | 0.200 | down |
| 11 | SYT5 | Synaptotagmin-5 | O00445 | 0.626 | 0.006 | 0.200 | up |
| 12 | CD151 | CD151 antigen | P48509 | -1.094 | 0.008 | 0.222 | down |
| 13 | KRT2 | Keratin, type II cytoskeletal 2 epidermal | P35908 | -0.912 | 0.010 | 0.259 | down |
| 14 | CXCL12 | Stromal cell-derived factor 1 | P48061 | -0.642 | 0.011 | 0.259 | down |
| 15 | KRT6B | Keratin, type II cytoskeletal 6B | P04259 | -0.909 | 0.011 | 0.259 | down |
| 16 | SLK | STE20-like serine/threonine-protein kinase | Q9H2G2 | -0.939 | 0.012 | 0.259 | down |
| 17 | IGKV4-1 | Immunoglobulin kappa variable 4-1 | P06312 | 1.017 | 0.013 | 0.259 | up |
| 18 | IGKV1-8 | Immunoglobulin kappa variable 1-8 | A0A0C4DH67 | 1.605 | 0.014 | 0.259 | up |
| 19 | SLPI | Antileukoproteinase | P03973 | -0.551 | 0.014 | 0.259 | down |
| 20 | APOA5 | Apolipoprotein A-V | Q6Q788 | -0.674 | 0.017 | 0.294 | down |
| 21 | APMAP | Adipocyte plasma membrane-associated protein | Q9HDC9 | -0.746 | 0.019 | 0.297 | down |
| 22 | LYZ | Lysozyme C | P61626 | -0.616 | 0.020 | 0.297 | down |
| 23 | RBP4 | Retinol-binding protein 4 | P02753 | 0.787 | 0.020 | 0.297 | up |
| 24 | APOE | Apolipoprotein E | P02649 | -0.696 | 0.022 | 0.318 | down |
| 25 | KRT78 | Keratin, type II cytoskeletal 78 | Q8N1N4 | -0.971 | 0.024 | 0.330 | down |
| 26 | RAB40B | Ras-related protein Rab-40B | Q12829 | 0.491 | 0.027 | 0.344 | up |
| 27 | GPRC6A | G-protein coupled receptor family C group 6 member A | Q5T6X5 | -0.619 | 0.027 | 0.344 | down |
| **Continued Supplementary Table 1. Characteristics of differentially expressed proteins between the GDM and control groups** | | | | | | | |
| **No.** | **Gene** | **Protein name** | **Uniprot ID** | **log_2_FC** | ***P* value** | **q value** | **Direction** |
| 28 | KRT6A | Keratin, type II cytoskeletal 6A | P02538 | -0.828 | 0.031 | 0.385 | down |
| 29 | ACTN1 | Alpha-actinin-1 | P12814 | -0.800 | 0.033 | 0.385 | down |
| 30 | LTF | Lactotransferrin | P02788 | -0.528 | 0.034 | 0.385 | down |
| 31 | ENO1 | Alpha-enolase | P06733 | -0.500 | 0.037 | 0.414 | down |
| 32 | VEGFA | Vascular endothelial growth factor A, long form | P15692 | 0.464 | 0.039 | 0.419 | up |
| 33 | KRT5 | Keratin, type II cytoskeletal 5 | P13647 | -0.692 | 0.045 | 0.467 | down |
| 34 | TTR | Transthyretin | P02766 | 0.937 | 0.046 | 0.467 | up |
| FC, fold change. Differentially expressed proteins were defined as those with \|log₂FC\| > log₂(1.2) and P ≤ 0.05 in the GDM group relative to the control group.  q value, Benjamini–Hochberg false discovery rate (FDR)-adjusted values. | | | | | | | |

| **Supplementary Table 2. Selection frequency of candidate proteins across the 39 outer iterations of leave-one-matched-pair-out nested LASSO** | | |
| --- | --- | --- |
| **Protein** | **Frequency**  **(n = 39 matched pairs)** | **Selection rate (%)** |
| APOL1 | 39 | 100.0 |
| GPRC6A | 39 | 100.0 |
| PRSS1 | 39 | 100.0 |
| RFC1 | 39 | 100.0 |
| SAA4 | 39 | 100.0 |
| TCEAL6 | 39 | 100.0 |
| ACTN1 | 37 | 94.9 |
| LGALS3BP | 37 | 94.9 |
| PCYOX1 | 32 | 82.1 |
| TGFBI | 30 | 76.9 |
| IGKV4-1 | 28 | 71.8 |
| ORM2 | 25 | 64.1 |
| SHROOM3 | 25 | 64.1 |
| C2CD2L | 23 | 59.0 |
| LYZ | 20 | 51.3 |
| TTR | 20 | 51.3 |
| APCS | 12 | 30.8 |
| SERPINA6 | 10 | 25.6 |
| SPP2 | 10 | 25.6 |
| SLPI | 9 | 23.1 |
| IGLV2-8 | 8 | 20.5 |
| AGT | 4 | 10.3 |
| **Continued Supplementary Table 2. Selection frequency of candidate proteins across the 39 outer iterations of leave-one-matched-pair-out nested LASSO** | | |
| **Protein** | **Frequency**  **(n = 39 matched pairs)** | **Selection rate (%)** |
| CXCL12 | 4 | 10.3 |
| LTF | 3 | 7.7 |
| MASP2 | 3 | 7.7 |
| SLK | 3 | 7.7 |
| SYT5 | 2 | 5.1 |
| APOC2 | 1 | 2.6 |
| BPIFB1 | 1 | 2.6 |
| C1QTNF4 | 1 | 2.6 |
| CD151 | 1 | 2.6 |
| LCAT | 1 | 2.6 |
| RAB40B | 1 | 2.6 |
| SERPINA7 | 1 | 2.6 |
| VEGFA | 1 | 2.6 |
| Only proteins selected in at least one outer iteration are shown; the remaining 309 of 344 quality-controlled proteins were not selected in any of the 39 outer iterations.  All 344 quality-controlled proteins entered the nested feature-selection procedure without prior differential-expression filtering. Selection count represents the number of outer iterations in which a protein had a non-zero LASSO coefficient. | | |

| **Supplementary Table 3. Internal discrimination of the nested proteomic feature-selection framework** | | | |
| --- | --- | --- | --- |
| **Validation framework** | **Outer resampling unit** | **AUC** | **95% CI** |
| Nested leave-one-pair-out CV | 39 matched pairs | 0.953 | 0.894–0.992 |
| The outer loop held out one complete matched case–control pair per iteration. All feature selection and LASSO tuning were performed within the training pairs using all 344 quality-controlled proteins. The AUC was calculated from pooled out-of-fold predictions, and the 95% CI was estimated by bootstrap resampling of the 39 matched pairs. Because upstream QC correction/normalization was performed before cross-validation and incorporated GDM group information, this estimate should be interpreted as exploratory internal discrimination rather than as a completely leakage-free or externally validated predictive performance estimate. | | | |

| **Supplementary Table 4. Matched-pair permutation analysis of the nested LASSO AUC** | | | |
| --- | --- | --- | --- |
| **Observed AUC** | **Permutation replicates** | **Permuted AUC ≥ observed** | **Empirical P** |
| 0.953 | 1,000 | 0 | 0.001 |
| Within each permutation, labels were randomly exchanged or retained within matched pairs and the downstream 344-protein nested LASSO procedure was repeated. The empirical P value used the +1 correction. Because upstream QC correction/normalization incorporated GDM group information before permutation, this analysis is interpreted as a conditional empirical check of the downstream modeling procedure rather than a test of complete absence of information leakage. | | | |

| **Supplementary Table 5. Sensitivity analyses excluding potentially contaminating or technically related proteins** | | | |
| --- | --- | --- | --- |
| Protein set | No. of proteins | AUC | Within-pair concordance |
| All proteins | 344 | 0.953 | 0.974 |
| Minus contaminants | 328 | 0.916 | 0.923 |
| Minus contaminants + Ig variable-region proteins | 288 | 0.918 | 0.949 |
| AUCs were estimated using the fully nested leave-one-matched-pair-out LASSO procedure, with all 344 quality-controlled proteins entered into the feature-selection framework without prior differential-expression filtering. Sensitivity analyses were performed after sequential removal of potentially contaminating/technically related proteins and immunoglobulin variable-region proteins. These analyses used the same matched-pair cross-validation framework and were intended to assess the robustness of the observed internal discrimination. The reported AUCs represent exploratory internal performance and do not constitute externally validated predictive performance. | | | |

Supplementary Figure 1. Characteristics of total proteins between different groups and trend clustering information. (A) PCA plot of QC, GDM, and control (CTR) samples. Each point represents one individual sample; colors: QC (red), CTR (blue), GDM (yellow). Ellipses denote 95% confidence regions. (B) OPLS-DA score plot distinguishing GDM from control groups. (C) Validation of the OPLS-DA model by 200-permutation test.

Supplementary Figure 2. First-trimester DEP PPI and hub-protein analysis. (A) PPI network. (B) Top 7 hub proteins ranked by MCC. (C) Exploratory ROC curves for the 7 hub proteins. ROC results are descriptive and were not cross-validated or externally validated.
